# Leishmaniasis: Vector–Host–Pathogen Interactions in Health and Disease

**DOI:** 10.3390/tropicalmed10070199

**Published:** 2025-07-17

**Authors:** Pedro Cecilio, Manuela da Silva Solcà, Nuno Santarém

**Affiliations:** 1 Vector Biology Section, Laboratory of Malaria and Vector Research, National Institute of Allergy and Infectious Diseases, National Institutes of Health, Rockville, MD 20852, USA; 2 Instituto Gonçalo Moniz—Fundação Oswaldo Cruz, R. Waldemar Falcão, 121-Candeal, Salvador 40296-710, Bahia, Brazil; 3 Escola de Medicina Veterinária e Zootecnia, Universidade Federal da Bahia, Av. Milton Santos, 500-Ondina, Salvador 40170-110, Bahia, Brazil; 4 Institute for Research and Innovation in Health (i3S), University of Porto, 4200-135 Porto, Portugal

Leishmaniases comprise a group of diseases caused by protozoan parasites belonging to different species of the genus *Leishmania*; of note; in humans, leishmaniasis presents as a spectrum of clinical syndromes, with the visceral, cutaneous, and mucosal forms being the most prominent [1]. According to the World Health Organization, over one billion individuals reside in regions where leishmaniasis is endemic, and are thus at risk of contracting the disease [2]. Worryingly enough, each year approximately 30,000 new cases of visceral leishmaniasis and more than 1 million new cases of cutaneous leishmaniasis are estimated to occur [2]. Importantly, *Leishmania* parasites are transmitted to humans through the bite of an infected female phlebotomine sand fly—a small hematophagous insect [1]. This fact alone makes the control of leishmaniasis a challenging task. Additionally, there is a strong correlation between leishmaniasis and socioeconomic vulnerability, including factors such as malnutrition, poor housing, and immunosuppression [3,4,5]. Therefore, it is of critical importance to improve our understanding of the epidemiological dynamics and etiopathogenesis of this vector-borne neglected tropical disease, toward its future effective control.

In this Special Issue of *Tropical Medicine and Infectious Diseases* on “**Leishmaniasis: Vector–Host–Pathogen Interactions in Health and Disease”**, we have invited original contributions describing fundamental breakthroughs related to sand flies, *Leishmania* parasites, and/or animal reservoirs, as well as those focused on applied approaches for the control of leishmaniasis. A total of 43 authors contributed with six publications, all original research articles.

Three studies from Europe, Africa, and South America focused on sand fly vectors, aiming to better understand the ecoepidemiology of the transmission of different sand fly-borne agents including *Leishmania* spp. parasites and phleboviruses [6,7,8]. Amaro et al. report results from an entomological survey carried out in the south of Portugal (Algarve) from May to October 2018 [6]. They were not only able to trap four of the five sand fly species known to occur in Portugal, but also to identify sand flies infected with either *Leishmania donovani* complex parasites (likely *L. infantum*) or two different phleboviruses (Massilia virus together with a potentially new phlebovirus) [6]. Overall, these authors re-attest that *Phlebotomus perniciosus* sand flies are the primary vector of *Leishmania* parasites in the region, and conclude that these parasites are co-circulating with phleboviruses in the same sand fly population [6]. Of note, no sand fly infected with both agents simultaneously was collected in this study, reproducing the findings reported in similar studies performed in different Mediterranean countries [9,10]. These results may imply that either there is an incompatibility between *Leishmania* parasites and phleboviruses in sand flies (with potential implications for disease control) [11], or that co-infection is just a rare scenario. Further studies are needed to understand which of the above possibilities is true.

Jemberie et al. also report results from an entomological survey, but in the north of Ethiopia, with emphasis on the vectors of cutaneous leishmaniasis in urban versus rural settings [7]. The authors also trapped five different sand fly species, three within the genus *Sergentomyia*, and two within the genus *Phlebotomus*, from February to August 2021 [7]. Of note, among the latter species, the authors briefly highlight *P. orientalis* for being recorded at a high elevation (2400 m; the highest ever reported in Ethiopia, according to them) [7]. Furthermore, in the context of a spatiotemporal analysis, the authors highlight a significant link between the preference for habitat and seasonality, in this case, in the context of *P. longipes*, vectors of *L. aethiopica*-caused cutaneous leishmaniasis in the region [7]. Particularly, these sand flies seem to mostly be found outdoors, in caves and peridomestic ecotypes in the dry season, while they seem to mostly be found indoors during the wet season [7]. Of note, the latter include blood-fed, infected specimens, suggesting the potential for indoor transmission [7], in line with the hypothesized by others [12,13].

Ferreira de Souza et al. are the last group of authors reporting results from an entomological survey, specifically in sylvatic versus urban/peridomestic settings in Minas Gerais, Brazil, from September 2012 to February 2014 [8]. In this South American study, the diversity of sand fly species captured was much greater than that reported in the above studies, with 24 species recorded. Strikingly, among these, nine species were infected with *Leishmania* parasites; notably, three *Leishmania* species, *L. braziliensis*, *L. amazonensis*, and *L. guyanensis*, circulate in the studied locales, although conclusions on the vector–parasite pairings responsible for the transmission of these agents cannot be withdrawn, as parasite identification was based on DNA amplification [8]. As a differentiation factor of this study, the authors also analyzed the source of blood in the context of the blood-fed specimens collected [8]. Interestingly, clear differences in the blood sources identified in sand flies collected from urban/peridomestic versus sylvatic settings were reported, with humans and domesticated animals (particularly chickens and pigs) being the main source of blood in the former, and wild animals, such as agoutis, armadillos, and hedgehogs being the main source of blood in the latter [8]. Interestingly, the authors comment on the possibility that these wild mammals are possible reservoirs of *Leishmania*, although in a speculative manner, as no *Leishmania* spp.–blood source association was revealed [8]; as they admit, more studies are needed to confirm their hypothesis.

All in all, these three studies stress the unexploited potential of entomology-based eco-epidemiological analyses, particularly in areas with active parasite transmission. More than to incriminate vectors, they may be essential to, e.g., identify sand fly breeding sites, establish vector behavior including expected alterations secondary to seasonal changes, and even point to potential animal reservoirs that altogether will allow the understanding/prediction of local transmission dynamics, and inform the development and implementation of tailored control approaches.

One other study, the fourth published in the frame of this research topic, was also ecology/epidemiology-based, but focused on the disease itself: human visceral leishmaniasis (HVL) in South America. Miranda et al. report the spatial distribution of HVL and its relationship with socioeconomic, environmental, and public health policy variables in four mesoregions of the state of Pará, Brazil, from 2011 to 2022 [14]. Overall, the authors conclude that HVL remains a major public health concern in the region due to a combination of multiple factors, not only climate-related, but also of a socioeconomic nature. These include environmental degradation, deforestation, disordered population growth and a consequent large migratory flow of construction workers, other changes in human circulation patterns and an increase in the circulation of domestic animals without zoonotic control, and a number of poverty-related factors such as a lack of environmental sanitation, nutritional insecurity, difficult access to health care services, and precarious housing conditions [14]. Such studies alone are important to inform policy makers and allow them to develop and implement more efficient and effective public policies. That said, the integration of such studies with other eco-epidemiological ones, for example, centered on vectors (as those highlighted above) and reservoirs, would be of paramount importance, ultimately contributing to a real understanding of disease transmission, and consequently, to the development and implementation of effective control measures.

The last two studies published in the frame of our research topic take us from the field to the lab. Quite different from each other, one explores more fundamental questions related to host–pathogen interactions [15], while the other has a more translational inclination, with emphasis on the treatment of leishmaniasis considering the potential emergence of drug resistance [16]. In the former study, Rodríguez-González et al. investigate the role of ERK and Akt in the maturation of monocyte-derived Dendritic Cells (moDCs) infected with metacyclic *L. mexicana* promastigotes [15]. Using both Akt- and ERK-specific inhibitors in the context of an in vitro system, the authors show a coordinated action between Akt and ERK during the infection of moDCs by *L. mexicana* parasites, which delays cell maturation (as per the decrease in the expression of CD86 but not MHC-II molecules) and decreases the secretion (but not expression) of IL-12. Overall, the authors conclude that the regulation of Akt is crucial during the infection process of moDCs by *L. mexicana* and speculate a possible role of such regulation by this *Leishmania* species (and particularly of different strains) in the induction of localized versus diffuse cutaneous leishmaniasis (LCL and DCL, respectively). This speculation is worthy of investigation in the future, especially in an integrated approach that considers immune responses (and the multiple cell and molecular mediators) as a whole.

Last but not least, building on the important concepts of drug repurposing and combination therapy, Melcón-Fernández et al. report the potential of pyrvinium pamoate (PyP), an FDA-approved anthelmintic with known antiparasitic and antiproliferative activities, for the treatment of leishmaniasis [16]. Traditionally used against pinworm infections, PyP exploitation as a repurposed agent for the treatment of leishmaniasis presents some limitations related to its bioavailability [17]; however, these may be overcome using combination approaches with other reference anti-leishmanial drugs. In fact, the authors demonstrated, using both axenic amastigotes and intra-macrophage infections, that PyP, when combined with clinically used drugs such as miltefosine and paromomycin (PMM), presented synergistic antiparasitic effects (particularly in association with PMM), without significant toxicity. Importantly, this combination strategy not only enhances the overall efficacy at lower drug concentrations of PyP, indirectly improving its pharmacokinetic profile, but also presents a strategy to mitigate treatment failure due to both the emergence of drug resistance and the adverse effects commonly associated with the currently used monotherapies. Overall, this work effectively highlights two crucial aspects of drug development for neglected diseases like leishmaniasis: the much-needed drug development “short cut” of drug repurposing and the exploration of the therapeutic value of combination regimens.

Overall, the main goal of our Special Issue, of compiling studies with a broad focus on leishmaniasis and vector–host–pathogen interactions in health and disease, has been accomplished. All in all, the studies published in this Special Issue highlight the complexity of the field, as well as some of the many questions that remain unanswered. Finding answers to these questions, or in other words, continuing to deepen our knowledge of leishmaniasis as a whole, is the only alternative we have toward the development of infallible strategies for the effective control of this spectrum of diseases (even in the absence of elimination of the etiological agents).
